# The usefulness of the Deep Learning method of variational autoencoder to reduce measurement noise in glaucomatous visual fields

**DOI:** 10.1038/s41598-020-64869-6

**Published:** 2020-05-12

**Authors:** Ryo Asaoka, Hiroshi Murata, Shotaro Asano, Masato Matsuura, Yuri Fujino, Atsuya Miki, Masaki Tanito, Shiro Mizoue, Kazuhiko Mori, Katsuyoshi Suzuki, Takehiro Yamashita, Kenji Kashiwagi, Nobuyuki Shoji

**Affiliations:** 1 Department of Ophthalmology, Graduate School of Medicine and Faculty of Medicine, The University of Tokyo, Tokyo, 113-8655 Japan; 2 Seirei Hamamatsu General Hospital, Shizuoka, 432-8558 Japan; 3 Seirei Christpther University, Shizuoka, 433-8558 Japan; 4 Department of Ophthalmology, Graduate School of Medical Sciences, Kitasato University, Kanagawa, 252-0374 Japan; 5 Department of Ophthalmology, Osaka University Graduate School of Medicine, Osaka, 565-0871 Japan; 6 Department of Ophthalmology, Shimane University Faculty of Medicine, Shimane, 693-8501 Japan; 7 Division of Ophthalmology, Matsue Red Cross Hospital, Shimane, Japan; 8 Department of Ophthalmology, Ehime University Graduate School of Medicine, Ehime, 791-0295 Japan; 9 Department of Ophthalmology, Kyoto Prefectural University of Medicine, Kyoto, 602-8566 Japan; 10 Department of Ophthalmology, Yamaguchi University Graduate School of Medicine, Yamaguchi, 755-0046 Japan; 11 Department of Ophthalmology, Kagoshima University Graduate School of Medical and Dental Sciences, Kagoshima, 890-0075 Japan; 12 Department of Ophthalmology, University of Yamanashi Faculty of Medicine, Yamanashi, 409-3898 Japan

**Keywords:** Translational research, Data processing

## Abstract

The aim of the study was to investigate the usefulness of processing visual field (VF) using a variational autoencoder (VAE). The training data consisted of 82,433 VFs from 16,836 eyes. Testing dataset 1 consisted of test-retest VFs from 104 eyes with open angle glaucoma. Testing dataset 2 was series of 10 VFs from 638 eyes with open angle glaucoma. A VAE model to reconstruct VF was developed using the training dataset. VFs in the testing dataset 1 were then reconstructed using the trained VAE and the mean total deviation (mTD) was calculated (mTD_VAE_). In testing dataset 2, the mTD value of the tenth VF was predicted using shorter series of VFs. A similar calculation was carried out using a weighted linear regression where the weights were equal to the absolute difference between mTD and mTD_VAE_. In testing dataset 1, there was a significant relationship between the difference between mTD and mTD_VAE_ from the first VF and the difference between mTD in the first and second VFs. In testing dataset 2, mean squared prediction errors with the weighted mTD trend analysis were significantly smaller than those form the unweighted mTD trend analysis.

## Introduction

Glaucoma causes irreversible and progressive visual field (VF) damage and is the second leading cause of blindness in the world^1^. Treatment decisions are guided by interpreting VF defects, however, VF sensitivity measurements fluctuate in both the short^2^ and long-term^3^. Measurement noise is considerable even when reliability indices are good^4,5^, which hampers the accurate estimation of VF progression^6^.

Machine learning consists of discriminative and generative models. Variational Autoencoders (VAEs) are a type of deep learning method that allow powerful generative models of data^7,8^. A VAE consists of an encoder, a decoder, and a loss function. The input data is first processed using a neural network (the encoder) and represented as a probability density in a latent space; the encoder is responsible for learning a mapping from the raw input data to a low dimensional latent space. The decoder is also a neural network and it reconstructs the data from the probability density; the decoder is responsible for learning the inverse mapping that reconstructs the original input. The parameters in the encoder and decoder are optimized so that the loss function (calculated as the difference between the input data and the reconstructed data) becomes minimal. VAEs have demonstrated remarkable generative capacity and modeling flexibility, especially with image data. Indeed VAEs have been used for various purposes, such as anomaly detection (for example, in Electrocardiograms^9^), clustering, and in particular, noise filtering^10^. Consequently, VAEs may be useful to filter VF noise and improve the reproducibility of VF measurements. Indeed, we have recently demonstrated its usefulness in improving the structure-function relationship between VF sensitivity and optical coherence tomography-measured nerve fiber layer thickness in glaucoma^11^. The first purpose of the present study was to investigate this hypothesis.

VF trend analyses, such as those in the Humphrey Guided Progression Analysis™ (GPA) software on the Humphrey Field Analyzer (HFA, Carl Zeiss Meditec, Dublin, CA, USA) and PROGRESSOR® (Medisoft Ltd., London, UK), are commonly used at the clinical setting^12^. Mean deviation (MD) trend analysis^13–15^ is probably the most frequently used method to assess the speed of glaucomatous VF progression, whereby ordinary least-squares linear regression (OLSLR) is applied on the VF measurement over time^16^. MD is an averaged value of VF damage across the entire VF, and as a result, is not sensitive to detect focal VF progression. Consequently, point-wise linear regression (PLR)^13–15^ is more useful than an MD trend analysis to detect early VF progression^17–21^, however, an assessment of progression in the entire VF cannot be obtained with PLR^22^. We recently reported that applying the binomial test to the point-wise linear regression results (an approach we call ‘binomial PLR’) enabled a more reliable and accurate diagnosis of progression in the whole field compared to an MD trend analysis^23,24^. The second purpose of the current study was to investigate whether reconstructing VFs using a VAE is useful to improve the accuracy and reliability of MD trend analysis and also binomial PLR.

## Methods

All protocols were reviewed and approved by the review board of the University of Tokyo, Kitasato University, Osaka University Graduate School of Medicine, Shimane University Faculty of Medicine, Matsue Red Cross Hospital, Ehime University Graduate School of Medicine, Kyoto Prefectural University of Medicine, Yamaguchi University Graduate School of Medicine, Kagoshima University Graduate School of Medical and Dental Sciences, and University of Yamanashi Faculty of Medicine. Patients gave written consent for their information to be stored in the hospital database and used for research, otherwise the study protocols did not require that each patient provide written informed consent, based on the Japanese Guidelines for Epidemiologic Study 2008 regulations, issued by the Japanese Government. Instead study participants were notified the protocol posted at the outpatient clinic. The studies complied with the tenets of the Declaration of Helsinki.

### Training dataset

All VF data recorded at the University of Tokyo Hospital between 2002 and 2018 was included in the training dataset (‘Tokyo dataset’). The data consisted of 82,433 VFs from 16,836 eyes of 9,139 subjects. All the VFs were measured using the HFA (24–2 or 30–2 Swedish Interactive Threshold Algorithm, SITA, standard program). Reliability criteria applied were: fixation losses (FL) less than 33%, false-positive (FP) responses less than 33% and false-negative (FN) rate less than 33%.

### Testing dataset 1 (analysis with test-retest dataset)

This dataset included VF measured using the HFA (24–2 or 30–2 SITA standard program) twice within three months from one hundred and four eyes of 104 open angle glaucoma patients, prospectively recruited at the glaucoma clinic in the University of Tokyo Hospital.

All patients enrolled in the study fulfilled the following criteria, similarly to our previous study:^10^ (1) no disease other than glaucoma that can cause VF damage; (2) at least two VF experience prior to the inclusion of this study; (3) glaucomatous VF defects defined as three or more contiguous total deviation points at p < 0.05, or two or more contiguous points at p < 0.01, otherwise a 10 dB difference across the nasal horizontal midline at two or more adjacent points, or MD worse than −5 dB^25^; (4) visual acuities at least 6/6. Only reliable VFs were used in the analysis, defined as: fixation losses less than 33%, false-positive responses less than 33% and false-negative rate less than 33%.

### Testing dataset 2 (trend analysis testing)

This dataset consisted of 638 eyes of 417 patients with primary open glaucoma with ten VF records excluding an initial VF, with no ocular comorbidities other than glaucoma, that may affect the VF. The inclusion and exclusion criteria of this dataset were described elsewhere^26^. That is, all VFs were recorded using the HFA (SITA standard 24–2 or 30–2 test pattern with a Goldmann size III target), derived from ten institutes in Japan. Two test points correspond to the blind spot were excluded from the analyses. When a VF was measured using the 30–2 test pattern, only the 52 test points overlapping with the 24–2 test pattern were used to derive the mean total deviation value (mTD). Oher inclusion, exclusion and reliability criteria were identical to those in testing dataset 1.

#### Pre-processing visual fields with a variational autoencoder

**Figure 1:**
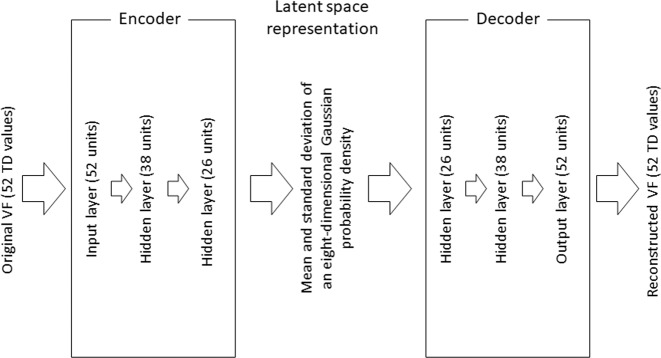
Structure of the variational autoencoder. The encoder is a 1-layer neural network consisting of 52 units. This isconnected to 1 hidden layer with 26 units. The information is then represented as the mean and standard deviation of eight Gaussian distributions in the latent space. Next, the decoder reconstructs the 52 TD values through 1 hidden layer with 26 units and a 1-layer neural network of 52 units. VAE: variational autoencoder, TD: total deviation, VF: visual field.

The structure of the VAE model is shown in Fig. 1. This was built using the training dataset. The encoder is a 1-layer neural network consisting of 52 units (for each of the 52 TD values). This encoder is connected to 2 hidden layers consisting of 38 and 26 units, and is then represented by the mean and standard deviation of an eight-dimensional Gaussian probability density in the latent space. The decoder reconstructs the 52 TD (TD_VAE_) values through a further 2 hidden layers and 1 output layer, which represents the reconstructed VF. This VAE model was optimized by maximizing the sum of the negative reconstruction loss, which is derived from the difference between the input VFs and reconstructed VFs and the Kullback–Leibler divergence between the distributions. mTD_VAE_ was calculated as the mean of 52 the TD_VAE_ values. In the VAE calculation, TD values were scaled to between 0 and 1 (before encoder), and then re-scaled back (after decoder) to the original values.

### Statistical analysis

#### Analysis with test-retest dataset

‘Test’ VFs in testing dataset 1 were reconstructed using the trained VAE. Then the difference between mTD values in the first VF (the ‘test’ VF) and the VAE-reconstructed first VF (the ‘test’ VF) was calculated. In addition, the difference between mTD values in the first VF (the ‘test’ VF) and in the second VF (the ‘retest’ VF) was calculated. The relationship between these two difference values was investigated.

#### mTD trend analysis

Using testing dataset 2, progression measured over all ten VFs (VF1–10) was regarded as a surrogate for true progression. The consistency of mTD trend analysis results was evaluated using the following measures, following our previous reports^23,24^:

1. Proportion of both progressing (PBP) was calculated as a surrogate measure for true-positive rate; i.e., where progression was significant in VF1-10 and also in shorter VFs (from VF1-9 to VF1-3).
2. Proportion of both not progressing (PBNP) was calculated as a surrogate measure for true negative rate; where progression in the complete series of VFs (VF1-10) was deemed “not significant”, and progression also “not significant” in shorter subsets of VFs (from VF1-9 to VF1-5).
3. Proportion of inconsistent progression (PIP) was calculated as a surrogate measure for the false positive rate; classification based on the shorter series of VFs (from VF1-9 to VF1-5) was judged to be “significant” but progression in the complete series of VFs (VF1-10) was “not significant.”

Further, mTD trend analysis was carried out using mTD values from the 1st to the 3rd VFs (VF1-3) of each patient, and the mTD values of the 10th VF test were predicted. The same procedure was carried out using the mTD values in longer series: VF1-4, VF1-5, VF1-6, VF1-7, VF1-8 and VF1-9, and the mTD values of 10th VFs were predicted every time.

In OLSLR, the regression line is decided so that the sum of squared residuals in the regression becomes minimum. In contrast, in a weighted linear regression analysis, the regression line is decided so that the sum of squared weighted residuals becomes minimum. To investigate the usefulness of the VAE for mTD trend analyses, the absolute difference between mTD and mTD_VAE_ values were calculated for each VF in testing dataset 2 and a weighted mTD trend analysis (mTD_VAE_ trend analysis) was performed using the difference as a weight in the regression (calculated as 1/absolute difference between mTD and mTD_VAE_ values). Then, similarly to the standard mTD trend analysis, PBP, PBNP, PIP values and also prediction accuracy were calculated using the mTD_VAE_ trend analysis and compared to those with the unweighted mTD trend analysis.

#### binomial PLR

The detailed calculation of the binomial PLR method is described in our previous report^23,24^. As detailed in our previous reports^23,24^, the assumption in PLR is that VF damage progresses linearly over time, similarly to the MD trend analysis^27–29^, where the null hypothesis was that the slope of VF progression was equal to 0. With this null hypothesis, slope p-values of coefficient from the linear regression can vary between the values of 0 and 1, where the numbers of test points with a p-value less than an arbitrary value would follow the binomial distribution. When this null hypothesis was rejected, a slope coefficient of zero is considered unlikely a result of random chance. In the current study, following our previous reports^22–24^, the significance of the entire VF progression was assessed using the four cut-off p values of 0.025, 0.05, 0.075, and 0.1. To represent these four p-values, the median p value was used^30,31^. A VF sequence was regarded as “significant” when the p-value calculated with the binomial PLR was <0.025; otherwise, it was “not significant.” Using this approach, PBP, PBNP, PIP, and the time to first detect a significant progression were calculated, similarly to the MD trend analysis.

Using the PBP, PBNP and PIP summary measurements, the accuracy of the weighted binomial PLR (binomial PLR_VAE_) was compared where the weight values were calculated as (1/absolute difference between TD and TD_VAE_ values). In addition, the number of VFs required to detect significant progression for the first time was calculated for each method. The sensitivity of each method to detect progression was assessed using Kaplan-Meier survival analysis and compared using the logrank test.

## Results

### Testing dataset 1 (analysis with test-retest dataset)

**Figure 2:**
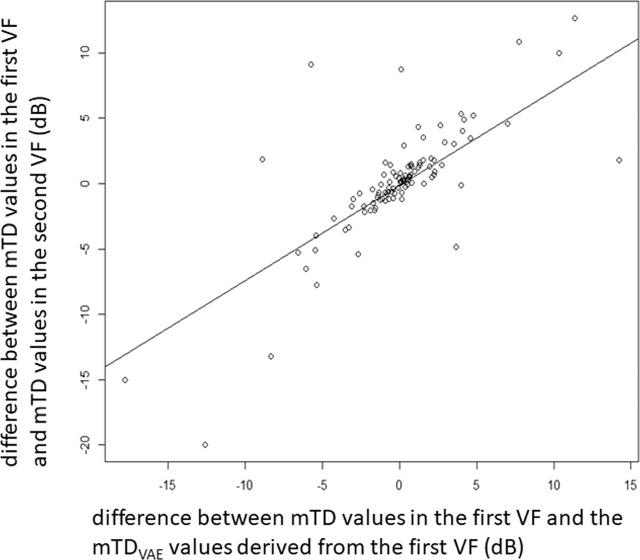
The relationship between the difference between mTD values in the first VF and the mTD_VAE_ values derived from the first VF and the difference between mTD values in the first VF and mTD values in the second VF. There was a significant relationship between the two values (R = 0.76, p < 0.001). mTD: mean total deviation, VAE: variational autoencoder.

Demographic summary data of testing dataset 1 is shown in Table 1. The mTD value in the second VF was significantly related both with the mTD value in the first VF (R = 0.83, p < 0.001, linear model) and mTD_VAE_ derived from the 1st VFs (R = 0.84, p < 0.001). There was a significant positive relationship between the difference between mTD values in the first VF and the mTD_VAE_ values derived from the first VF and the difference between mTD values in the first VF and mTD values in the second VF (R = 0.76, p < 0.001, Fig. 2). A significant relationship was not observed for FL (p = 0.81), FP (p = 0.55) or FN (p = 0.53) in the first VF.

**Table 1:** Subjects demographics in Testing dataset 1.

variable	value
Number of eyes	104
Number of subjects	104
Age, mean ± SD, y	62.0 ± 0.05
mTD of 1st VF, mean ± SD, dB	−9.8 ± 7.4
mTD of 2nd VF, mean ± SD, dB	−9.7 ± 7.3
FL of 1st VF, mean ± SD, %	4.9 ± 6.4
FL of 2nd VF, mean ± SD, %	5.0 ± 5.9
FP of 1st VF, mean ± SD, %	3.7 ± 5.4
FP of 2nd VF, mean ± SD, %	4.4 ± 6.3
FN of 1st VF, mean ± SD, %	2.9 ± 4.5
FN of 2nd VF, mean ± SD, %	3.1 ± 5.2

SD: standard deviation, mTD: mean total deviation, VF: visual field, FL: fixation loss, FP: false positive, FN: false negative.

### Testing dataset 2 (mTD trend analysis)

**Figure 3:**
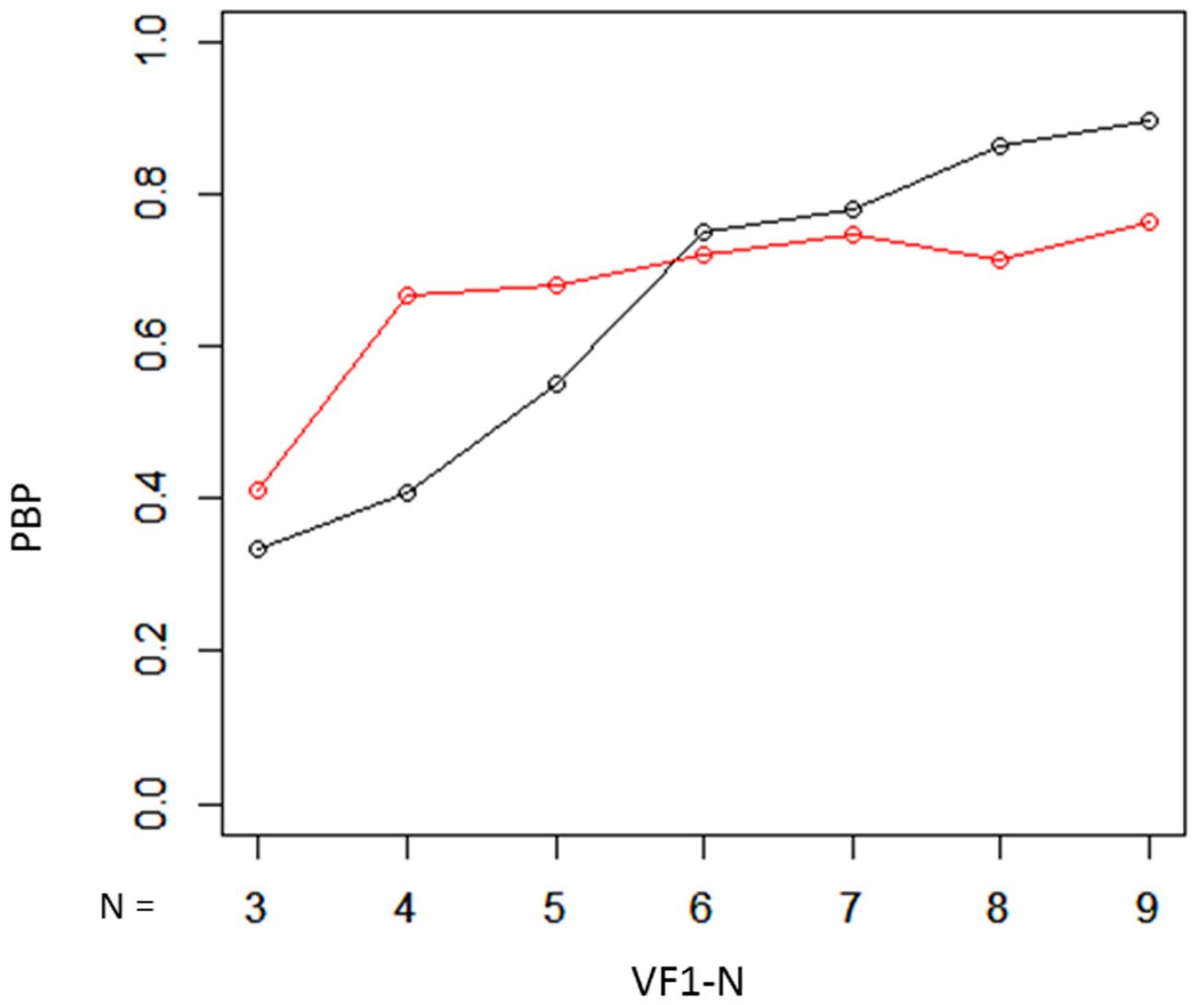
PBP values with unweighted mTD trend analysis and weighted mTD_VAE_ trend analysis. There was no significant difference in the PBP values of the two methods. PBP: probability both progressing, mTD: mean total deviation, VAE: variational autoencoder.

Demographic summary data of testing dataset 2 is shown in Table 2. Baseline mTD, follow-up period between VF1 and VF10, and mTD progression rate were − 6.9 ± 6.3 [Mean ± Standard Deviation] dB, 5.4 ± 1.1 years, and − 0.26 ± 0.46 dB/year, respectively. There was a significant relationship between the mTD and mTD_VAE_ (p < 0.001, linear mixed model where random effects were subject and number of VF).The PBP values with the standard unweighted mTD trend analysis and the weighted mTD_VAE_ trend analysis are presented in Fig. 3. PBP values were 0.33, 0.41, 0.55, 0.75, 0.78, 0.87, and 0.90 from VF1-3 to VF1-9 with the mTD trend analysis, respectively, whereas they were 0.41, 0.67, 0.68, 0.72, 0.75, 0.71, and 0.76, respectively, with the mTD_VAE_ trend analysis. There was no significant difference in the PBP values of the two methods (P = 0.14, paired Wilcoxon test).

**Table 2:** Subjects demographics in Testing dataset 2.

variable	value
Number of eyes	636
Number of subjects	415
Age, mean ± SD, y	54.7 ± 11.8
Eyes, R:L	307:329
mTD at the baseline, mean ± SD, dB	−6.9 ± 6.3
Follow-up, mean ± SD, y	5.4 ± 1.1
mTD progression rate, mean ± SD, dB/y	−0.26 ± 0.46
FL, mean ± SD, %	4.4 ± 5.2
FP, mean ± SD, %	2.1 ± 2.5
FN, mean ± SD, %	3.3 ± 4.9

SD: standard deviation, mTD: mean total deviation, FL: fixation loss, FP: false positive, FN: false negative.

**Figure 4:**
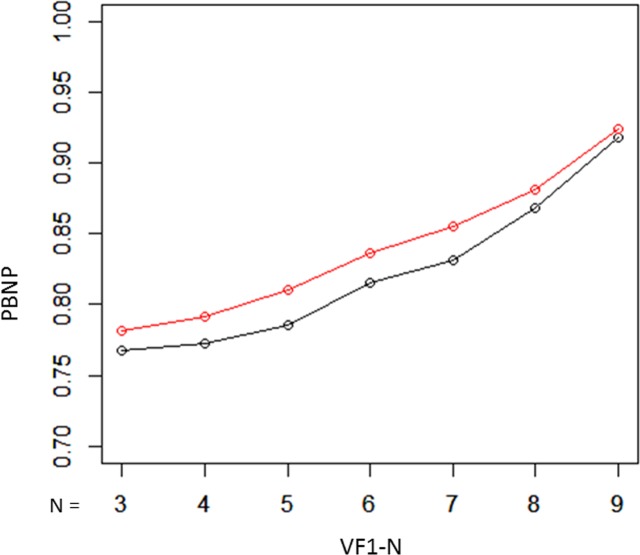
PBNP values with unweighted mTD trend analysis and weighted mTD_VAE_ trend analysis. PBNP values with the weighted mTD_VAE_ trend analysis were significantly higher than those of the unweighted mTD trend analysis. PBNP: probability both not progressing, mTD: mean total deviation, VAE: variational autoencoder.

The PBNP values with the unweighted mTD trend analysis and the weighted mTD_VAE_ trend analysis, are presented in Fig. 4. These values were 0.77, 0.77, 0.79, 0.82, 0.83, 0.87, and 0.92 from VF1-3 to VF1-9 with the mTD trend analysis, respectively, whereas they were 0.78, 0.79, 0.81, 0.84, 0.86, 0.88, and 0.92, respectively, with the mTD_VAE_ trend analysis. There was a significant difference in the PBNP values of the two methods (P = 0.016, paired Wilcoxon test).

**Figure 5:**
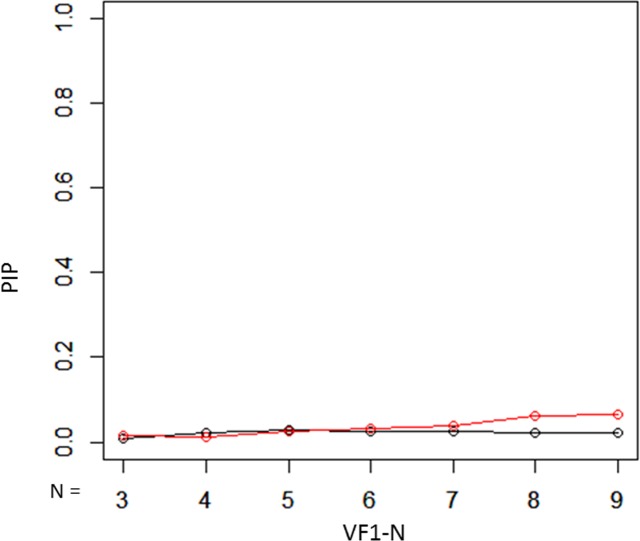
PIP values with unweighted mTD trend analysis and weighted mTD_VAE_ trend analysis. There was no significant difference in the PBP values of the two methods. PIP: probability inconsistent progression, mTD: mean total deviation, VAE: variational autoencoder.

The PIP values with the unweighted mTD trend analysis and weighted mTD_VAE_ trend analysis, are presented in Fig. 5. These values were 0.0096, 0.021, 0.028, 0.024, 0.026, 0.022, and 0.023 from VF1-3 to VF1-9 with the mTD trend analysis, respectively, whereas they were 0.016, 0.011, 0.024, 0.033, 0.038, 0.061, and 0.064, respectively, with mTD_VAE_ trend analysis. There was no significant difference in the PIP values of the two methods (P = 0.16, paired Wilcoxon test).

**Figure 6:**
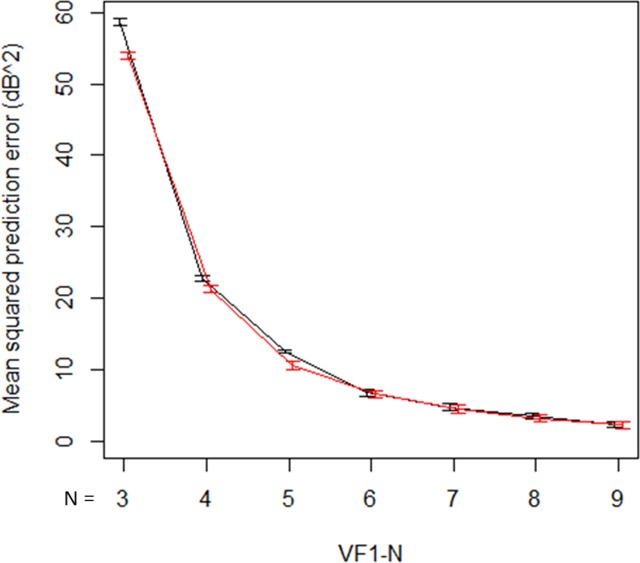
Prediction errors with unweighted mTD trend analysis and weighted mTD_VAE_ trend analysis. Black bar shows the prediction errors with unweighted mTD trend analysis, whereas red bar shows weighted mTD_VAE_ trend analysis. There was a significant difference in these values of the two methods. mTD: mean total deviation, VAE: variational autoencoder.

Figure 6 shows the comparison of mean squared prediction errors between the unweighted mTD trend analysis and the weighted mTD_VAE_ trend analysis. The errors were 58.7, 22.7, 12.6, 6.8, 4.7, 3.4, and 2.3 from VF1-3 to VF1-9 with the unweighted mTD trend analysis, respectively, whereas they were 53.0, 19.8, 11.0, 6.5, 4.6, 3.2, and 2.3, respectively, with the mTD_VAE_ trend analysis. There was a significant difference in errors from the two methods (P = 0.031, paired Wilcoxon test).

### Binomial PLR

**Figure 7:**
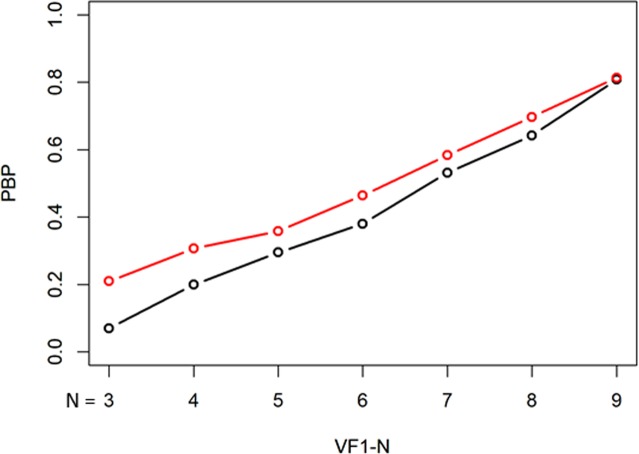
PBP with unweighted binomial PLR and weighted binomial PLR_VAE_. There was not a significant difference in the values of PBP between unweighted binomial PLR and weighted binomial PLR_VAE_. Black bar shows the PBP values with binomial PLR, whereas red bar shows weighted binomial PLR_VAE_. There was a significant difference in these values of the two methods. PBP: probability both progressing, PLR: point-wise linear regression, VAE: variational autoencoder.

**Figure 8:**
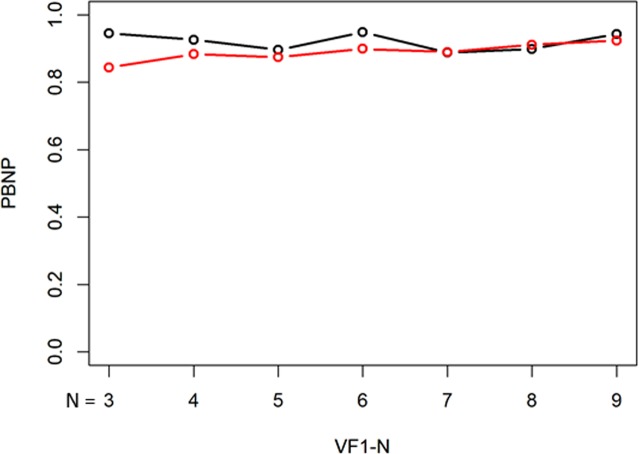
PBNP with unweighted binomial PLR and weighted binomial PLR_VAE_. Black bar shows the PBP values with binomial PLR, whereas red bar shows weighted binomial PLR_VAE_. The values of PBNP with weighted binomial PLR_VAE_ were significantly higher than those with unweighted binomial PLR. PBNP: probability both not progressing, PLR: point-wise linear regression, VAE: variational autoencoder.

**Figure 9:**
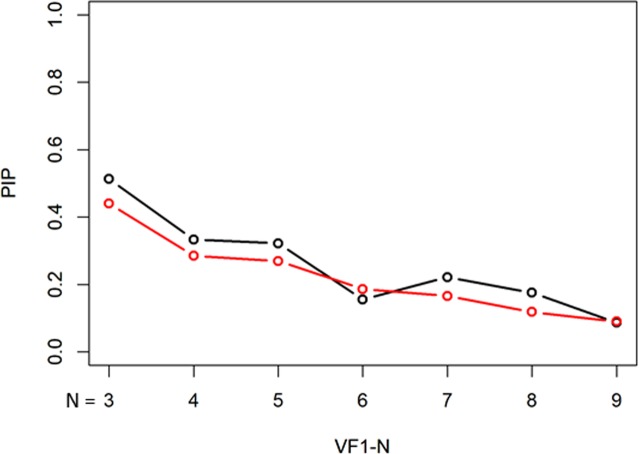
PIP with unweighted binomial PLR and weighted binomial PLR_VAE_. There was not a significant difference in the values of PIP between unweighted binomial PLR and weighted binomial PLR_VAE_. Black bar shows the PIP values with binomial PLR, whereas red bar shows weighted binomial PLR_VAE_. There was a significant difference in these values of the two methods. PIP: probability inconsistently progression, PLR: point-wise linear regression, VAE: variational autoencoder.

**Figure 10:**
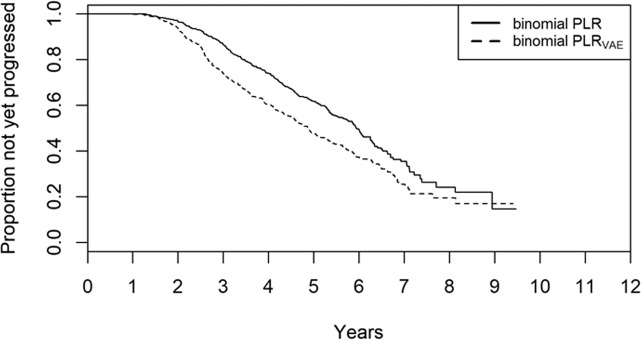
The Kaplan-Meier survival analysis with binomial PLR and binomial PLR_VAE_. The results of the Kaplan-Meier survival analysis with binomial PLR and binomial PLR_VAE_ trend analysis are shown. PLR: point-wise linear regression, VAE: variational autoencoder.

Figures 7, 8, and 9 show the comparisons of PBP, PBNP, and PIP values between the unweighted binomial PLR and weighted binomial PLR_VAE_. The PBP values with binomial PLR ranged from 0.07 with VF1-3 to 0.81 with VF1-9, whereas those with binomial PLR_VAE_ were between 0.21 with VF1-3 and 0.81 with VF1-9. The PBNP values with binomial PLR ranged from 0.89 with VF1-7 to 0.95 with VF1-3, whereas those with binomial PLR_VAE_ were between 0.84 with VF1-3 and 0.92 with VF1-8 and VF1-9, respectively. The PIP values with binomial PLR ranged from 0.088 with VF1-9 to 0.51 with VF1-3, whereas those with binomial PLR_VAE_ were between 0.090 with VF1-9 and 0.44 with VF1-3, respectively. There was not a significant difference in the values of PBNP and PIP (p = 0.078 and 0.078, paired Wilcoxon test), whereas the values of PBP with binomial PLR_VAE_ were significantly higher than those with binomial PLR (p = 0.016, paired Wilcoxon test). Kaplan-Meier survival analysis and the logrank test indicated that the binomial PLR_VAE_ detected significantly more progressing eyes than the binomial PLR, (P < 0.0001) (Fig. 10). The time to classification of progression with each method was: 6.8 ± 2.9 (mean ± SD) years with mTD trend analysis, 4.3 ± 1.6 years with binomial PLR_VAE_, and 4.7 ± 1.5 years with binomial PLR.

## Discussion

In the current study, a VAE model was developed using 82,433 VFs from 16,836 eyes of 9,139 subjects. The usefulness of this method to improve the reproducibility of mTD measurements, and accuracy of trend analyses was investigated. VF reproducibility, in the form of test-retest mTD, was better using the VAE-derived measurement (mTD_VAE_). The accuracy of mTD trend analysis and binomial PLR was enhanced using the mTD_VAE_; further, the sensitivity of binomial PLR was improved using mTD_VAE_.

VF data are inherently associated with measurement noise. In the current study, it was suggested that it is beneficial to consider mTD_VAE_ in addition to mTD itself to predict the mTD value in the retest VF. In particular, when mTD_VAE_ took a larger value than the value of mTD in the first VF, mTD in the second VF tended to be larger than that in the first VF (suggesting the measurement may be under-estimated). Conversely when mTD_VAE_ derived from the first VF took a smaller value than the value of mTD, mTD value in the second VF tended to be smaller than that in the first VF (suggesting the measurement may be over-estimated).

Visual field measurement noise has a considerable effect on the accuracy of trend analyses^6^. In the current study, the average mTD progression rate was −0.26 ± 0.46 dB/year. From real world clinics, Heijl *et al*. reported a VF progression rate of −0.80 dB/year, in 583 patients with open angle glaucoma, where the average baseline MD value was −10.0 dB (median)^32^. In 587 patients with glaucoma, De Moraes *et al*. reported a −0.45 dB/year VF progression rate when the baseline MD value was equal to −7.1 dB (mean)^33^. As shown by the analysis of the test-retest data in the current study, mTD_VAE_ was related to mTD in the second VF after an adjustment for mTD in the first VF. This implies that the accuracy of mTD trend analyses may be improved by considering the difference between mTD_VAE_ and mTD. Indeed, the current results suggested that applying a weighted linear regression using these differences as weights yielded more accurate predictions compared to the unweighted approach (see Fig. 6).

We previously reported that applying least absolute shrinkage and selection operator (LASSO) in linear regression resulted in much more accurate prediction error than the conventional mTD trend analysis^34^. Similarly, we also reported that Variational Bayesian Linear Regression, in which the mTD trend analysis was optimized by considering the temporal and spatial VF defect patterns, enabled more accurate predictions of VF progression compared to the conventional mTD trend analysis^35,36^. The magnitude of increase in prediction accuracy with the mTD_VAE_ trend analysis is smaller than that observed in these previously reported models. However, accuracy may be further improved by combining the current approach with these other regression models.

There have been previous studies which suggested the usefulness of non-linear regression, instead of liner regression, in VF trend analysis^17,37–40^. However we previously investigated the usefulness of the application of such non-linear regression approaches (exponential, quadratic, and logistic regressions, as well as robust regression models) in VF trend analysis^41^. The experiment setting was very similar to that in the current study: future VF was predicted using prior (shorter) VF sequences. As a result, it was suggested no improvement of the prediction error was obtained by any method, compared to the conventional ordinary least squares linear regression. In addition, there is another non-negligible drawback of the application of the non-linear regression model at the clinical settings; significance of the obtained non-linear curve cannot be calculated. Thus, although it may be of interest to further investigating the usefulness of applying the current approach to such non-linear regression models, the clinical usefulness would be limited.

There was not a significant difference between PBP (Fig. 3) and PIP (Fig. 4) values for the standard mTD trend analysis and the proposed mTD_VAE_ trend analysis. This suggests that these methods have similar sensitivity and false positive rates when diagnosing progression. On the other hand, PBNP value with the mTD_VAE_ trend analysis, however, was significantly higher than that with the conventional mTD trend analysis (Fig. 5), suggesting the new approach has better specificity. We previously reported that applying the binomial test to PLR resulted in improved PBP and PIP values compared to standard mTD trend analysis. We further investigated whether using a weighted PLR (with weights equal to the differences between TD and TD_VAE_ values at each test point) is beneficial in binomial PLR. Sensitivity (Fig. 7) and the false positive rate (Fig. 8) were not significantly different between the two methods, however significantly higher PBNP values were obtained with binomial PLR_VAE_ compared to binomial PLR (Fig. 9). Furthermore, the sensitivity to detect progression was significantly better with binomial PLR_VAE_ than with binomial PLR (Fig. 10).

In the current study, there was not a significant positive relationship between the difference between mTD and the mTD_VAE_ values, and FL, FP and FN. FL, FP and FN are the indices currently used to assess the reliability of measured VF. More specifically, FL, FP, FN is thought to indicates test reliability and vision fixation, “trigger-happy” patients, and inattention during an examination^25,42–46^. While some past studies have reported on the usefulness of these indices^47,48^, more recent studies have suggested their limitations; for instance, FLs can also result from the mislocalization of the blind spot^49^ and fixational instability can be found even in well trained observers^43,50^. A high FN rate is reported to be associated with the amount of field loss as well as threshold reproducibility^4^. The VF noise estimated by the difference between mTD and the mTD_VAE_ values cannot be explained by these VF reliability indices, but we speculate that this is because of these limitations of these reliability measures. Another possible approach would be further investigating this issue using a microperimetry with retinal tracking, such as MP-3 (Nidel Co.Ltd., Aichi, Japan), because more accurate assessment of VF can be conducted preventing the effect of eye movement (mis-location)^51^.

One of the limitations of the current study is a lack of results from the HFA 10-2 test. Recent studies have revealed that it is recommended to measure the HFA 10-2 VF in addition to the HFA 24-2^52–57^. In addition, damage to this area of the VF is more directly associated with patients’ vision related to the quality of life^58,59^ A future study should be attempted shedding light on the usefulness of VAE in the HFA 10-2 test. In addition, various spatial filter methods, such as^60^, have been reported which are other possible approach to reduce VF noise. It would be of interest to investigate the usefulness of them compared to VAE in a future study. In addition, generative adversarial network (GAN)^61^ is further another possible deep learning approach to reduce noise in VF. In general, GAN generates images which look more natural by human beings compared to VAE, however VF is not a material to be recognized the shape by human beings, so this merit may and may not be observed in VF. It would be of interest to compare the usefulness of VAE and GAN in a future study.

In conclusion, we developed a method to reconstruct the VF measurement using a deep learning method. The approach appears to be useful to predict MD value in the retested VF and also to improve the reliability of MD trend analyses and also binomial PLR.
